# The BXD21/TyJ recombinant inbred strain as a model for innate inflammatory response in distinct brain regions

**DOI:** 10.1038/s41598-020-70213-9

**Published:** 2020-08-05

**Authors:** Caridad López-Granero, Beatriz Ferrer, Alessandra Antunes dos Santos, Angel Barrasa, Michael Aschner

**Affiliations:** 1 Department of Molecular Pharmacology, Albert Einstein College of Medicine, Bronx, NY 10461 USA; 2 Departamento de Psicología y Sociología, Universidad de Zaragoza, Campus Ciudad Escolar, 44003 Teruel, Spain

**Keywords:** Cytokines, RNA, PCR-based techniques, Genetic models

## Abstract

Oxidative stress and inflammatory cytokines affect the human brain, increasing the risk for mood and cognitive disorders. Such risk might be selective to brain-specific regions. Here, we determined whether BXD recombinant inbred (RI) mice strains are more suitable than C57BL/6J mice for the understanding of the relationship between antioxidant response and inflammatory responses. We hypothesized that inflammatory responses could be independent of antioxidant response and be inherent to brain-specific regions. This hypothesis will be addressed by the analyses of mRNA expression. We explored, at 7-months-of-age, the innate activation of proinflammatory cytokines (tumor necrosis factor alpha (TNFα) and interleukin 6 (IL-6), as well as Kelch-like ECH-associating protein 1 (Keap1), nuclear factor erythroid 2 related factor 2 (Nrf2) and glutathione peroxidase 1 (Gpx1) mRNA in both male and female BXD84/RwwJ RI, BXD21/TyJ RI and control strain (C57BL/6J mice). We report that: (1) The cerebellum is more sensitive to antioxidant response in the BXD21/TyJ RI strain; (2) The cerebellum, hippocampus and striatum show increased levels of cytokines in the BXD21/TyJ RI strain; (3) The BXD RI strain has lower brain weight relative to control strain (C57BL/6 mice). In conclusion, our novel data show the utility of the BXD21/TyJ RI strain mice in offering mechanistic insight into Nrf2’s role in the inflammatory system.

## Introduction

Central nervous system (CNS) dysfunction is frequently accompanied by oxidative stress and inflammatory responses^1^. Oxidative damage and inflammatory cytokines influence brain function and result in increased risk for mood, behavioral and cognitive disorders^2–4^. Thus, the general hypothesis is that antioxidant defenses and inflammatory cytokines are key elements in CNS pathologies^5^ and psychiatric disorders^3,4,6,7^.

Several studies have revealed the mechanism by which continued oxidative stress can lead to chronic inflammation, which, in turn, could mediate most chronic diseases^8^. The disruption of the inflammatory and oxidative stress pathways is associated with multiple neurotoxic exposures^9–11^. Oxidative stress acts activating a variety of transcription factors, including Nrf2. Upon oxidative stress generation, Nrf2 dissociates from Kelch-like ECH-associating protein 1 (Keap1), and translocates into the nucleus where it binds to the antioxidant response element (ARE), and initiates antioxidant gene transcription thus restoring cellular redox homeostasis^12–15^. Activation of these transcription factors can lead to the expression of different genes, including those for inflammatory cytokines and anti-inflammatory molecules^8^. Indeed, Nrf2 is essential for protection against oxidative stress and it has also been shown to attenuate inflammation^16^.

Consistent with these observations, published data have corroborated dysregulation of inflammatory and oxidative systems both in behavioral and psychiatric disorders as a consequence of altered Nrf2 pathway functioning^7,15^. Neuroinflammatory processes are established sequalae of Redox imbalances^17^. Hence, the importance of investigations into the relationship between nrf2 and cytokines. In contrast to the widely held view that Nrf2 suppresses inflammation through redox control, Kobayashi and collaborators^16^ have suggested that Nrf2 inhibits proinflammatory cytokine gene expression. Whatever the cause of the inflammatory response, an adaptive change in the inflammatory system may provide short-term benefits or it can become maladaptive if the stressor persists chronically^18^. According to Reuter and collaborators^8^, two stages of inflammation take place, acute and chronic. The former is of short duration. If the inflammation persists for a longer time, the second stage of inflammation, or chronic inflammation, sets in predisposing the host to various chronic illnesses^8,19^.

Previously, we have demonstrated that certain inflammatory responses could be desirable in mitigating psychiatric disorders, such as depressive behavior. We have observed that the BXD21/TyJ strain exhibited lessened immobility time in the forced swim test, congruent with lessened depression-like behavior^20^. Concomitantly, we noted overexpression of cerebral cortex proinflammatory cytokines, (tumor necrosis factor alpha (TNFα) and interleukin 6 (IL-6)) in the absence of oxidative stress^20^.

Oxidative stress, as well as inflammatory responses, have been linked to numerous neuropathologies associated to specific brain areas^21,22^. Several studies have indicated that metal homeostasis and oxidative damage is brain region-dependent^23^. Others have evaluated regional preferences for cytokine-mediated brain reactions to endotoxemia (elevated inflammatory response), noting that the olfactory system, hippocampus and diencephalon were the most responsive^21^. However the results between studies have been inconsistent and the relationship between oxidative stress and inflammation in brain-specific regions has yet to be addressed. The aim of the present study was to evaluate whether antioxidant response and inflammatory processes are region-dependent in BXD RI line and C57BL/6J mice. The cerebellum, hippocampus and striatum were selected, given their involvement in major human neuropathologies^23^.

The genetic reference murine populations have been generated from a cross between wild-type (C57BL/6J) (B6) and DBA/2J mice (D2), and is referred to as the BXD RI lines. The lines were generated following a strategy of progressive intercrosses greater than 20 generations^24^. BXD RI strains have been proven invaluable in understanding the genetics of behavioral phenotypes, such as drug and alcohol addiction, stress, impulsivity, nociception and pain sensitivity, to name a few^24^. Here, we chose the BXD84/RwwJ RI and BXD21/TyJ strains given their diverse expression lof Nrf2 mRNA. In an earlier report, we have demonstrated low expression of Nrf2 mRNA in the BXD84/RwwJRI strain and its high expression in BXD21/TyJ RI strain at postnatal day 21^20^. Since the BXD RI mouse strains and Nrf2 might offer an optimal platform for relating genetic influences with environmental exposure outcomes, the understanding of the relationship between BXD RI mice and Nrf2 is essential.

Here, we tested the hypothesis that inflammatory responses might appear independent of antioxidant response in a region-specific manner. To address our hypothesis, we explored proinflammatory cytokine (TNFα and IL-6 mRNA), Keap1, Nrf2 and glutathione peroxidase 1 (Gpx1) innate mRNA levels in the two selected BXD RI strains. To our knowledge, there have been no studies addressing the relationship between innate inflammatory mediators and nrf2 levels in the BXD RI lines (high and low Nrf2 expressors) with emphasis on various brain regions, which might be susceptible to neurotoxicity.

## Experimental procedures

### Animals

Six-week-old mice from the BXD RI strains and control (C57BL/6) were purchased from the Jackson Laboratory (Bar Harbor, ME). Groups of three to five mice per cage were accommodated with a 12 h light/dark cycle and water and food were continuously available ad libitum. For the first 15 days the animals were habituated to conditions in the animal facility. Control mice (C57BL/6) and two BXD RI mouse strains BXD84/RwwJ RI and BXD21/TyJ RI were studied as noted above (N = 12 per strain and n = 6 per sex). Animals used in the study were not exposed to any treatment or experimental condition, allowing for the evaluation of innate mediators of inflammation and redox homeostasis.

All experiments were approved and carried out in accordance with the Institutional Animal Care and Use Committee (IACUC) at Albert Einstein College of Medicine (Bronx, NY).

### Tissue collection and structure extractions

Mice were sacrificed with the use of isoflourane as anesthesia at age of 7 months old^20^. The brain was extracted, dissected out, and the cerebellum, hippocampus and striatum were rapidly flash-frozen in liquid nitrogen. All the samples were stored at − 80 °C.

### Gene expression assay

Proinflammatory genes (TNFα and IL-6), as well as antioxidant genes (Keap1, Nrf2 and Gpx1) were analyzed by quantitative reverse transcription PCR (qRT-PCR).

### Gene expression assay by TaqMan method

Total RNA from the cerebellum, hippocampus and striatum was extracted with Trizol (Life Technologies) as previously described^20^. Briefly, chloroform was added to each sample. Next, samples were spun at 4 °C for 15 min at 15,000 revolutions/min. This was followed by precipitation with glycogen (Ambion) and isopropanol. Next, the upper phase was transferred to a new tube. Samples were maintained overnight at − 20 °C. The next day, the pellet was washed with ethanol (75%). The RNA isolated was mix with nuclease-free water (Ambion) and heated at 55 °C (for 10 min). RNA purity and concentration were analyzed with a spectrophotometer NANODROP 2000 (Thermo Scientific). cDNA synthesis was carried out with RNA and High Capacity cDNA Reverse Transcription Kit (Life Technologies). qRT-PCR (BioRad CFX96) was carried out with TaqMan Gene Expression Assay probes (LifeTechnologies). The housekeeping gene GADPH was used as a control using the comparative 2^–ΔΔCt^ method^25^. The following probes were used: Keap1 (assay ID Mm00497268_m1), Nrf2 (assay ID: Mm00477784m1); Gpx1 (assay ID: Mm00656767_g1), TNFα (assay ID: Mm00443259_g1) and IL-6 (assay ID: Mm00446190_m1).

### Statistics

SPSS software package was used for all statistical analyses and GraphPad PRISM 6.0 for completing the graphics. The accepted level of significance for all tests was set at p ≤ 0.05. Two-way analysis of variance (ANOVA) was used for the following dependent variables: Keap1, Nrf2, Gpx1, TNFα and IL-6 mRNA levels. As independent variables STRAIN (BXD84/RwwJ RI, BXD21/TyJ RI and control mice (C57BL/6) and SEX (female and male) were used. Post hoc analysis was performed with Bonferroni test.

## Results

### Body and brain weight results

#### Brain weights are reduced in the BXD84/WRwwJ and RI BXD21/TyJ strains at 7-months-of age compared to control (C57BL/6) mice in the absence of any treatment or experimental condition

**Figure 1:**
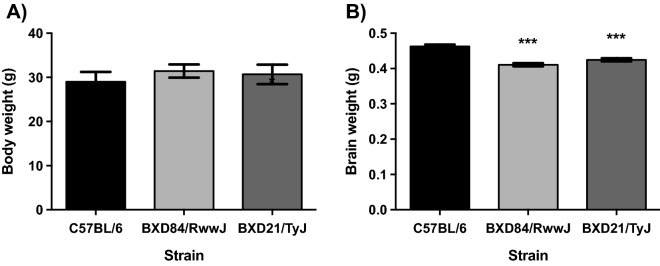
Mean (± SEM). Two-way analysis of variance (ANOVA) and Bonferroni post hoc test was used. (**A**) Body weight and (**B**) Brain weight in three selected strains: BXD84/RwwJ RI, BXD21/TyJ RI mice and C57BL/6 wild type as control group at 7-months-of-age (n = 12). (*) Statistical analyses indicate significant brain weight differences between BXD21/TyJ RI and BXD84/RwwJ RI strains and the control group (p ≤ 0.001).

ANOVA revealed no statistically significant differences in body weights between BXD84/RwwJ RI mice and BXD21/TyJ RI compared to controls mice at 7-months-of-age (Fig. 1A). However, brain weights showed statistically significant effects (Fig. 1B). The brain weights in BXD84/RwwJ (p = 0.000) and RI BXD21/TyJ RI (p = 0.000) mice were lower compared to controls mice (F_(2, 30)_ = 30.054; p = 0.000). Females and males from the three examined strains showed statistically indistinguishable body or brain weights.

### Gene expression in the cerebellum, hippocampus and striatum

At 7-months-of-age, we performed qRT-PCR to examine inflammatory and antioxidant responses in 2 BXD RI strains (BXD84/RwwJ RI and BXD21/TyJ RI mice) in the cerebellum, hippocampus and striatum.

### BXD21/TyJ RI mice display decreased levels of Nrf2 mRNA and overexpression of Gpx1 and IL-6 mRNA levels in the cerebellum

**Figure 2:**
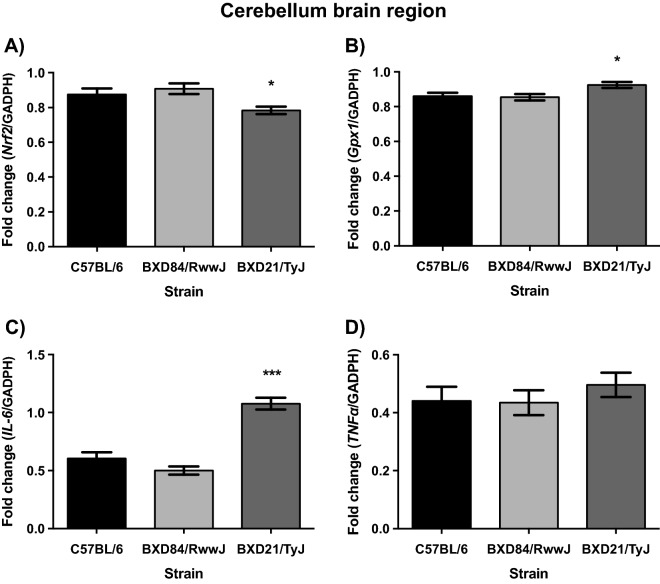
Mean (± SEM). Two-way analysis of variance (ANOVA) and Bonferroni post hoc test was used. Levels of Nrf2, Gpx1, IL-6 and TNFα mRNA in cerebellum brain region in three selected strains: BXD84/RwwJ RI, BXD21/TyJ RI mice and C57BL/6 wild type as control group at 7-months-of-age (n = 6–12). (**A**) Levels of Nrf2 mRNA grouping by strains. (**B**) Levels of Gpx1 mRNA grouping by strains. (**C**) Levels of IL-6 mRNA grouping by strains. (**D**) Levels of TNFα mRNA grouping by strains (*) and (***) Statistical analyses indicate significant differences between the BXD21/TyJ RI strain and the control group and BXD84/RwwJ RI strain (p ≤ 0.05) or (p ≤ 0.001).

Statistical analysis revealed a significant effect on Nrf2 mRNA levels on STRAIN (F_(2, 30)_ = 6.106; p = 0.006). The BXD21/TyJ RI strain displayed lower level of Nrf2 mRNA compared to control (p = 0.055) mice and BXD84/RwwJ RI mice (p = 0.006) (Fig. 2A). The analyses showed a main effect on STRAIN by SEX interaction (F_(2, 30)_ = 5.178; p = 0.012) in relation to Nrf2 in cerebellum. However, this main effect was lost in the post hoc analyses.

As for Keap1 mRNA levels, at 7-months-of-age, no statistically significant differences were noted between the experimental groups, neither in STRAIN by SEX interaction (F_(2, 30)_ = 0,938; p = 0.403).

In addition, analyses on Gpx1 mRNA levels revealed a significant effect on STRAIN (F_(2, 30)_ = 4.931; p = 0.014) but not on STRAIN by SEX interaction (F_(2, 30)_ = 1,559; p = 0.227). Increased Gpx1 mRNA levels were seen in BXD21/TyJ RI mice compared to controls and BXD84/RwwJ RI mice (p = 0.044 and p = 0.025 respectively) (Fig. 2B).

With respect to the neuroinflammatory response, statistical analyses showed an effect on STRAIN (F_(2, 30)_ = 47.836; p = 0.000). BXD21/TyJ RI mice displayed increased levels of IL-6 mRNA compared to controls (p = 0.000) and BXD84/RwwJ RI mice (p = 0.000) (Fig. 2C). TNFα mRNA levels were indistinguishable between the 3 strains (Fig. 2D). Analyses from proinflammatory genes did not showed any STRAIN by SEX interaction (F_(2, 30)_ = 0.592; p = 0.559; F_(2, 30)_ = 1.085; p = 0.351) in IL-6 and TNFα mRNA levels.

### BXD21/TyJ RI mice overexpress TNFα and IL-6 mRNA levels in the hippocampus

**Figure 3:**
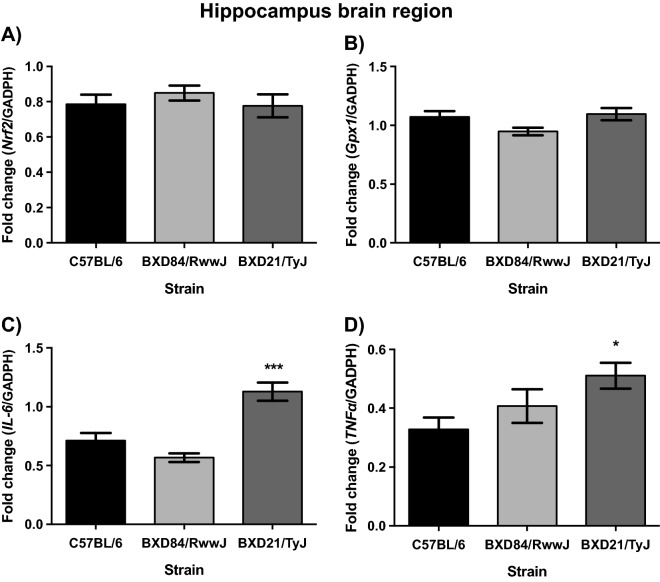
Mean (± SEM). Two-way analysis of variance (ANOVA) and Bonferroni post hoc test was used. Levels of Nrf2, Gpx1, IL-6 and TNFα mRNA in hippocampus brain region in three selected strains: BXD84/RwwJ RI, BXD21/TyJ RI mice and C57BL/6 wild type as control group at 7-months-of-age (n = 6–12). (**A**) Levels of Nrf2 mRNA grouping by strains. (**B**) Levels of Gpx1 mRNA grouping by strains. (**C**) Levels of IL-6 mRNA grouping by strains. (**D**) Levels of TNFα mRNA grouping by strains (*) and (***) Statistical analyses indicate significant differences between the BXD21/TyJ RI strain and the control group and BXD84/RwwJ RI strain (p ≤ 0.001) in (**C**) and statistical analyses showed significant differences between the BXD21/TyJ RI strain and the control group in (**D**) (p ≤ 0.05).

Data showed no differences in Keap1, Nrf2, and Gpx1 mRNA levels between, BXD84/RwwJ RI, BXD21/TyJ RI and controls mice. At 7-months-of-age, the tested strains showed similar levels of hippocampal Keap1, Nrf2 (Fig. 3A) and Gpx1 (Fig. 3B) mRNA levels. No statistical differences were noted for STRAIN by SEX interaction.

A significant statistical STRAIN effect was observed on TNFα (F_(2, 29)_ = 3.965; p = 0.030) and IL-6 mRNA levels (F_(2, 30)_ = 25.238; p = 0.000). Post hoc analyses shown augmented proinflammatory response in BXD21/TyJ RI compared to BXD84/RwwJ RI (p = 0.000) and controls (p = 0.000) for levels of IL-6 mRNA (Fig. 3C) and control for levels of TNFα mRNA (p = 0.026) (Fig. 3D). Females and males from the three strains displayed same levels of hippocampal antioxidant and proinflammatory genes (STRAIN by SEX interaction for IL-6 mRNA (F_(2, 30)_ = 2.268; p = 0.121) and for TNFα mRNA levels (F_(2, 30)_ = 0.894; p = 0.420).

### BXD21/TyJ RI mice overexpress IL-6 mRNA levels in the striatum

In striatum, ANOVA analyses exhibited no differences in Keap1, Nrf2, Gpx1 and TNFα mRNA levels between, BXD84/RwwJ RI, BXD21/TyJ RI and controls mice. At 7-months-of-age, all the strains showed similar levels of striatum Keap1, Nrf2 (Fig. 3A), Gpx1 (Fig. 3B) and TNFα (Fig. 3D) mRNA levels. Females and males from the three strains displayed the same levels of striatal antioxidant mRNA levels. However, there was no significant effect for STRAIN on TNFα mRNA levels, with the STRAIN by SEX interaction displaying a main effect (F_(2, 30)_ = 6.263; p = 0.005). However, post hoc analyses showed increased TNFα mRNA levels in females than in males in the three strains absent statistically significant differences.

Statistical analyses on neuroinflammatory levels revealed a significant effect on STRAIN (F_(2, 29)_ = 8.591; p = 0.001) with respect to IL-6 mRNA levels. BXD21/TyJ RI mice showed increased levels of IL-6 mRNA relative to BXD84/RwwJ RI and control (p = 0.006 and p = 0.002) (Fig. 3C). The STRAIN by SEX interaction in IL-6 mRNA did not reach statistical difference for the three strains and both sexes (F_(2, 30)_ = 0.261; p = 0.772).

## Discussion

**Figure 4:**
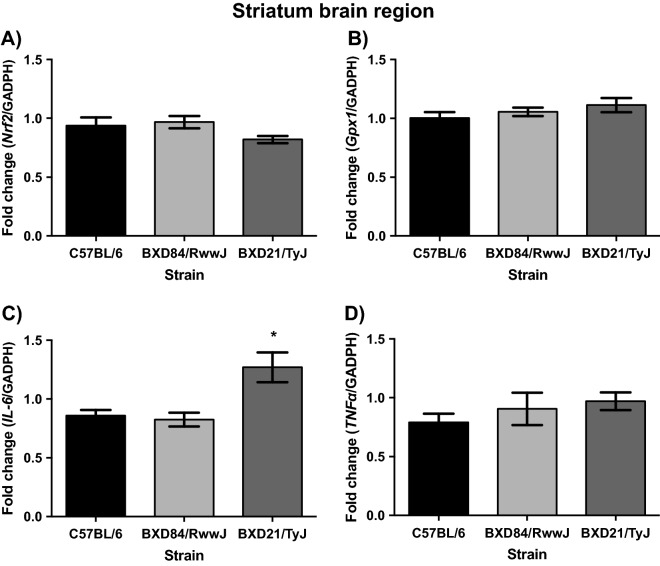
Mean (± SEM). Two-way analysis of variance (ANOVA) and Bonferroni post hoc test was used. Levels of Nrf2, Gpx1, IL-6 and TNFα mRNA in striatum brain region in three selected strains: BXD84/RwwJ RI, BXD21/TyJ RI mice and C57BL/6 wild type as control group at 7-months-of-age (n = 6–12). (**A**) Levels of Nrf2 mRNA grouping by strains. (**B**) Levels of Gpx1 mRNA grouping by strains. (**C**) Levels of IL-6 mRNA grouping by strains. (**D**) Levels of TNFα mRNA grouping by strains (*) Statistical analyses indicate significant differences between the BXD21/TyJ RI strain and the control group and BXD84/RwwJ RI strain (p ≤ 0.05).

Both, oxidative stress and inflammatory responses affect brain function and mediate the risk for behavioral alterations in psychiatric and neurologic pathologies^3–6^. To our knowledge, this is the first study to report the relationship between innate inflammatory and innate antioxidant responses in BXD RI strains in the cerebellum, hippocampus and striatum based on mRNA expression levels. We noted congruence between innate elevated levels of antioxidant response and increased levels of cytokines in the cerebellum in BXD21/TyJ RI strain (Fig. 2). Furthermore, all studied brain regions, cerebellum, hippocampus and striatum, showed an inflammatory profile (Figs. 2, 3 and 4), suggesting an innate inflammatory susceptibility in BXD21/TyJ RI mice. In the hippocampus and striatum we failed to note congruence between enhanced antioxidant and cytokine profiles. Thus inflammatory and antioxidant profiles within a single murine mouse strain are brain region-dependent. We highlight the great utility of the BXD21/TyJ RI mice as a model for studying innate inflammatory and antioxidant responses.

### BXD84/RwwJ RI and control mice do not show an innate change in the profile of inflammation and antioxidant response

Firstly, female and male BXD84/RwwJ RI and controls mice did not show any change in antioxidant level or proinflammatory cytokines profiles in any of the studied brain regions (Figs. 2, 3 and 4). Several authors have addressed Nrf2 expression levels under normal conditions absent experimental procedures^26^. Under such circumstances, Nrf2 has a short half-life of 10–30 min, with high turnover of Keap1, assuring Nrf2 basal levels remain low^27,28^. Considering that animals herein were not manipulated experimentally, the levels of Nrf2 and antioxidant should be at low basal levels. Consistent with this assertion, BXD84/RwwJ RI and controls failed to show increased innate antioxidant and inflammatory responses, in contrast to the BXD21/TyJ RI strain.

### Nrf2 mRNA levels in BXD21/TyJ RI mice

BXD21/TyJ RI mice showed lower cerebellar Nfr2 mRNA levels concomitant with increased levels of Gpx1 mRNA relative to BXD84/RwwJ RI and controls mice (both females and males showed the same pattern) (Fig. 2). At 7-months-of-age, we expected to see higher nrf2 mRNA levels in the BXD21/TyJ RI strain (based on previous pilot studies in our lab at PND 21). This discrepancy reinforces the idea of adaptive regulation or compensatory mechanisms in the Nrf2 system^29,30^ from PND 21 to 7 months-of-age. Indeed, Nrf2 levels can quickly vary in response to environmental alterations^15^ and tried to reach normal balance in Nrf2 levels (that is low levels) as an innate phenomenon.

### Is there correspondence between inflammatory and antioxidant responses in BXD21/TyJ RI mice?

Several studies have reported that continued oxidative stress leads to inflammation^8^. However, our results indicated correspondence only between inflammatory cytokines and antioxidant response by increasing the levels of Gpx1 mRNA in the cerebellum of BXD21/TyJ RI mice (Fig. 2). Some authors have highlighted the crucial role of antioxidant expression in preventing toxic effects^31,32^. In this sense, Gpx1 is one of the most relevant antioxidant capable of reacting against oxidative stress as a therapeutic factor^33^. The role of Gpx1 is to modulate cellular oxidant stress responses^31^. Gpx1 may be post- and transcriptionally upregulated as part of the cellular response to oxidative stress^31^ . The regulation of expression of GPx-1 has been shown to play a role in the development of many diseases such as cancer and cardiovascular disease, indicating the potential use of Gpx1 as a therapeutic^31^.

The congruence between antioxidant response and inflammatory cytokines was not seen in the hippocampus and striatum where the BXD21/TyJ RI mice showed elevated proinflammatory response absent altered antioxidant profiles (Figs. 2, 3 and 4). Supporting our results, other authors have noted that Nrf2-mediated inhibition of proinflammatory cytokine gene is independent of redox control^16^. However, these authors observed that Nfr2 inhibited expression of proinflammatory cytokine genes, suggesting that it was due to alternative mechanism to redox control^16^. Our result suggest that cytokine regulation might be dependent of Nrf2 function in BXD21/TyJ RI mice via Gpx1 mRNA as to maintain redox-balance in cerebellum, but not in the hippocampus and striatum. In these two-brain regions, the Nrf2 is likely mediated by alternative mechanisms, given the absence of overexpression of antioxidant genes, such as Gpx1, despite the presence of high cytokine levels.

### Relative to previous results in our research group

Here, we propose that the key to understand Nrf2’s role in the innate inflammatory system response might reside in the adaptive role of such response. In support of this notion, we have previously observed similar pattern of inflammatory cytokines response in cortex region with a protection against depression in the BXD21/TyJ RI strain without oxidative stress response^20^. In order to maintain normal homeostasis complex interactions occur between cytokines, inflammation, and the adaptive and innate responses^34^. In the BXD21/TyJ RI strain, the innate activation of cytokines observed herein concomitant with the short immobility time and thus reduced depression-like behavior^20^ provides additional impetus for studying novel antidepressants in a BXD RI animal model of innate inflammation.

### Are proinflammatory cytokines and antioxidant response region-dependent in BXD21/TyJ RI?

Our results suggest that the cerebellum (Fig. 2) is more sensitive to antioxidant response compared to other brain regions (hippocampus and striatum (Figs. 3 and 4 respectively). In contrast, other authors have noted that in the striatum oxidative damage was more pronounced than in the cerebellum, hippocampus, and hypothalamus^23^. However, this susceptibility to oxidative stress was noted in a tributyltin exposure model, a neurotoxin that induces oxidative injury^35^. Consistent with our results, Rammal and collaborators^17^ indicated discrepancy in redox homeostasis upon stress conditions in neuronal and glial cells in cerebellum. It is noteworthy that this study also found oxidative stress in the hippocampal region, where we failed to note antioxidant response. The discrepant may reside in the fact that they^17^ analyzed oxidative damage in neurons and glial cells, contrary to our study, where antioxidant levels were analyzed in homogenized tissue. Given that glial cells possess an antioxidative system defense^36,37^ further evaluation of glial-specific responses in BXD21/TyJ RI seems meritorious.

In addition the cerebellum, hippocampus and striatum are susceptible to cytokines (Figs. 2, 3 and 4), Elevated proinflammatory cytokines in these brain regions^21^ have been noted, establishing that the cortex, hippocampus, olfactory system, striatum, brain stem, diencephalon and cerebellum responded to lipopolysaccharide-induced systemic inflammation with altered cytokine profiles. The widespread nature of brain cytokine production appears also congruent with the characteristics of sepsis-associated encephalopathy^21^.

### Lower brain weight in the BXD RI strain

Unexpectedly, we found lower brain weight in the BXD RI strain (both 21/TyJ and 84/RwwJ) relative to controls mice (Fig. 1). The BXD RI lines have been generated by crosses among DBA/2J mice (D2) and C57BL/6J mice (B6)^24^. Adult C57BL/6J (B6) and DBA/2J (D2) mice body weights are similar, but the former have 37% heavier brains^38^. The same authors evaluated 20 different BXD RI strains derived from D2 and B6 inbred strain crossings to determine whether significant associations exist between brain and brain to body weight ratio, concluding that BXD RI mice have lower brain weight, consistent with our findings.

Brain size is a historical subject of interest where the small size has been associated with some kind of alteration^39,40^. Some studies have indicated a close relationship between proinflammatory cytokines and obesity related to overproduction of white adipose tissue^41^. Obesity might be associated with low-inflammation, which eventually is spread from tissue to the brain with an ensuing cognitive decline^42^. However, here we have demonstrated overproduction of inflammatory cytokines accompanied by lower brain weight in BXD RI strain. Previously, we have indicated that the inflammation might be a double-edged sword in the BXD RI strain^20^, the contribution to behavioral alterations and as effective therapeutic target via astrogliosis function^43^. The lesser brain weight observed could be indicating an attempt to regulate the inflammatory response in the BXD21/TyJ RI strain as a protective mechanism. This fact reinforces the idea of further investigations into the relationship between inflammatory mechanisms and microglia in the onset of brain disorders.

### Future directions

#### Determine the nature of microglial diversity and its relationship to cytokine responses in BXD21/TyJ RI mice brain regions

It is a well-known fact that microglia have important functions in the central nervous system (CNS)^44,45^. Microglia may participate in synaptic transmissions during development and can phagocytize during brain injury^45^. The role microglia adopts in each scenario can be context-^45^ and brain-region-dependent^46^. In addition, it has been established that microglia respond to IL-6 among other kind of cytokines. Microglia are capable of producing and reacting to the immune system via responsiveness to cytokines and their autoregulation^47^. Here, we have seen that the cerebellum, hippocampus and striatum are susceptible to cytokines in BXD21/TyJ RI. Thus, it would be relevant to address in future studies whether the relationship between microglia and cytokines is inherent to different mice strains, and whether BXD21/TyJ RI mice might offer an optimally suited model to understand this relationship.

#### Examine the intra- and cellular pathways involved in the relationship between inflammatory and antioxidant responses

It would be necessary to perform new studies in order to analyze the interaction of the innate immune response and other intracellular pathways, such as NFKB (nuclear factor KB). Indeed, it has been established that NFKB has the ability to modify mtDNA, resulting in heightened sensing by innate immune receptors^48^.

In the other hand, even when there are proteins involved in the Nrf2 signaling pathway preventing the formation of lipid peroxides, also other protein are direct targets of lipoxidation^49^. A number of reactive lipid species, including 4-HNE have been shown to activate nrf2 target gene expression though Keap1 function^50^.

New pathways studies are necessary in order to shed light on inflammatory and antioxidant responses associated with the effects inherent to our results.

## Conclusions

Our results suggest Nrf2 plays an important role in the inflammatory system. Our novel findings favor antioxidant profiles, via mRNA expression evaluation, is region- and strain-dependent manner. Furthermore, we suggest that inflammation may occur with independent of innate antioxidant response profiles, contrary to the widely accepted view that Nrf2 suppresses inflammation. Our findings suggest that (1) the cerebellum is more sensitive to antioxidant response in the BXD21/TyJ RI strain, (2) the cerebellum, hippocampus and striatum showed innate inflammation along with increased levels of cytokines in the BXD21/TyJ RI strain, (3) in the BXD21/TyJ RI strain Nrf2 plays an important role in mediating inflammation via alternative mechanism/s to antioxidant gene activation, and (4) in the BXD21/TyJ RI strain, this alternative innate mechanism might be related with adaptive brain function. Altogether, our results shed novel information on the potential for the BXD21/TyJ RI mouse strain as model to advance mechanistic understanding on the cross-talk between Nrf2 and innate inflammatory and redox regulation.
